# Susceptibility status and synergistic activity of DDT and Lambda-cyhalothrin on *Anopheles gambiae* and *Aedes aegypti* in Delta State, Nigeria

**DOI:** 10.1371/journal.pone.0309199

**Published:** 2024-08-29

**Authors:** Chioma C. Ojianwuna, Victor N. Enwemiwe, Eric Esiwo, Favour Mekunye, Ann Anidiobi, Treasure E. Oborayiruvbe

**Affiliations:** Department of Animal and Environmental Biology, Faculty of Science, Delta State University, Abraka, Nigeria; University of Ibadan Faculty of Science, NIGERIA

## Abstract

The detection of insecticide resistance in male mosquitoes has been treated with less importance in monitoring insecticide resistance spread in mosquitoes. There are no studies on the susceptibility and synergistic activity of DDT and lambda-cyhalothrin on male *Anopheles gambiae* and *Aedes aegypti* in Delta State, Nigeria. Even though studies have extensively reported resistance in female mosquitoes, the susceptibility of male mosquitoes to insecticide classes should be ascertained. In this study, we tested the susceptibility status and synergistic activity of DDT and Lambda-cyhalothrin on *An*. *gambiae* and *Ae*. *aegypti* in Delta State, Nigeria, in order to ascertain the level of resistance and knockdown. In addition, we modelled the knockdown time using Probit analysis model. WHO bioassay method was used to expose two days old adult mosquitoes to 4% DDT and 0.05% lambda-cyhalothrin. The results showed that *An*. *gambiae* mosquitoes exposed to DDT and lambda-cyhalothrin were confirmed resistant (61% and 53% respectively). However, pre-exposing the resistant mosquito population to piperonyl butoxide (4%) showed an increase in mortality to 90% (possible resistance) in DDT and 98% (susceptible) in lambda-cyhalothrin. *Ae*. *aegypti* mosquitoes exposed to DDT were susceptible (98%) while those exposed to lambda-cyhalothrin were confirmed resistant (87%) and this increased to complete mortality (100%) in PBO+lambda-cyhalothrin population. Furthermore, the results showed that the knockdown time (KDT_50_ and KDT_95_) in *An*. *gambiae* exposed to DDT was 39.5–71.2 minutes and 124.5–146.4 minutes respectively, while that of lambda-cyhalothrin was 33.0–81.8 minutes and 64.0–124.4 minutes respectively. In *Ae*. *aegypti*, KDT_50_ and KDT_95_ was 23.9 and 61.7minutes for DDT exposure whereas it was 5.6–15.3 minutes and 36.1–72.3 minutes for lambda-cyhalothrin exposure. It can be concluded that male *An*. *gambiae* mosquitoes exposed to the insecticides were resistant and the causes may be linked to certain resistant genes in the mosquitoes. The chances of transferring resistance are possible in wild species and molecular-based studies on the resistant gene in male mosquitoes as well as the tendencies of transfer are required to establish this focus.

## Introduction

Vector control interventions is a principal component of public health and remains the only key measures for reducing human diseases such as malaria, lymphatic filariasis, yellow fever, chikungunya and other several vectored diseases [1]. Vector control has acknowledged the innovative application of insecticides into nets, as commercial sprays and other vital materials, irradiation of male mosquito into sterility, microbial blockage of the salivary components using the bacterium, *Wolbachia*, entomopathogens either fungi or nematodes, botanical insecticide use in various forms, and the introduction of natural enemies of mosquitoes in their breeding sites [2–7]. These intervention measures are effective in various degrees. For instance, early instars of mosquitoes are preyed upon by a large number of aquatic organisms including fish as applied in India [8], amphibians in Kenya [9], copepods in Vietam [10], odonate young instars [11, 12], water bugs [13], and even larvae of predacious mosquito species [14]. More so, several studies have shown the larval mortalities of several insecticidal plants [15, 16]. In all, the use of recommended insecticides delivered in form of insecticide-treated nets (ITNs) and indoor residual spraying (IRS) stands out as the most resorted control [17, 18]. Other insecticide application measures such as the use of sprays, treatment of breeding sites, and integration into materials designated for human use form another basic management approach in curtailing mosquito population [19]. Nine classes of chemical insecticides including pyrethroids, carbamates, organophosphates, organochlorines, neonicotinoids, pyrroles, butenolides, juvenile hormone mimics and spinosyns recommended by World Health Organization Pesticides (WHOPES) are prequalified for use [20].

Mosquito resistance to one or more of these recommended insecticide classes is the significant issues discouraging the sustainable use of these insecticides. The prevailing resistance of mosquitoes have mandated the uproar not only for newer technologies targeting mosquitoes but also for the combination of recommended insecticides with other insecticide classes to boost the effectiveness and even the use of synergists (piperonyl butoxide: PBO) and integrated approaches [21, 22]. Studies conducted in Nigeria have so far reported mosquito resistance to recommended insecticides [21–26]. Mosquito resistance to insecticides has been suggestively linked to several factors related to harsh environmental conditions mostly occurring in Northern Nigeria, oil spills on breeding sites mostly common in the Niger-Delta regions, runoff of agricultural pesticide into mosquito breeding sites, incessant sprays of insecticide without adherence to manufacturers guidelines, escape of resistant species into the wild and many other associating factors [27]. Species complexes in the genera *Anopheles*, *Culex* and *Aedes* are the major groups targeted in control studies [22].

Mosquito population resurge after bypassing the resulting effects of insecticides by detoxification and numerous strategies. Thus, the wide search for alternative interventions and approaches. Even with the numerous studies on the resistance of female mosquitoes to several recommended insecticides [28, 29], the transfer of resistant genes to the offspring is lacking in literature and should be addressed. Likewise, studies on the resistance of male mosquitoes to recommended insecticides are lacking. The speculative role of male mosquitoes in the transfer of resistant genes to offspring should be looked into as a new focus in studies. The study should monitor the generation time for resistant gene transfer and spread. Male mosquitoes are considered irrelevant in most insecticide susceptibility tests, since they do not transmit any disease. Suggestively, the role they play in reproduction should not be underestimated. This argument is opened to further researching as the resistance of male mosquitoes to recommended insecticides has been neglected. DDT and commercial sprays made of pyrethroids are commonly used insecticides in Delta State, Nigeria. These insecticides are commonly used as household sprays and agricultural uses. Hence, the need for this study to determine the resistance of male *Anopheles* and *Aedes* mosquitoes to Dichlorodiphenyltrichloroethane (DDT) and lambda-cyhalothrin in Aniocha South Local Government Area of Delta State in order to quantify the resistance ratio with females. These mosquitoes were selected for the potential role they play in malaria and arboviral transmission in Ika North East an adjoining LGA to this location [22]. Thus, early detection of resistance would help in the design of intervention measure that would help target the vector and disease spread.

## Materials and methods

### Study area and mosquito collection

The mosquitoes for this study were collected from Ogwashi-uku (Latitude, 6.181614 and Longitude, 6.530310), Nsukwa (Latitude, 6.13968 and Longitude, 6.479910), Ejeme (Latitude, 6.017726 and Longitude, 6.357176) and Ubulu-uku (Latitude, 6.23327 and Longitude, 6.44843), Aniocha South Local Government Area, Delta State, Nigeria. *Anopheles* and *Aedes* mosquitoes were collected from different breeding habitats as highlighted by [30]. Mosquito populations were pooled in equal proportion to form replicates for the test. The testing was carried out in the Insectary of the Department of Animal and Environmental Biology, Delta State University, Abraka, Nigeria. WHO standard larval collecting dippers were used for larvae and pupal sourcing. Immature stages of mosquitoes were cultivated in larval trays in the insectary maintained at room temperature. Emerging adult mosquitoes were transferred to adult holding cages where sorting by sex was done using the antennae as a reference point. Pictorial keys of *Anopheles* and *Aedes* mosquitoes were explored from Coetzee [31] and Rueda [32] respectively. Male mosquitoes had brushy antennae and were kept in a separate holding cage for the experiment. Prior to the bioassay, the male mosquitoes were fed for 48 hours using 10% glucose solution.

### Insecticide resistance bioassay (IRA)

IRA was done using the WHO bioassay method [33]. One hundred male mosquitoes sectioned into twenty-five per replicate were kept in the holding tube for 1 hour before they were blown into exposure tubes containing DDT (4%) and lambda-cyhalothrin (0.75%) impregnated paper. Mortality readings were taken at intervals of 10, 15, 20, 30, 40, 50 and 60 minutes [33]. Mosquitoes were then carefully transferred back to the holding tubes and kept for 24hours, during which they were fed with 10% glucose solution. Readings after 24 hours were taken to ascertain whether mosquitoes were susceptible or resistant to the exposed insecticides. Two replicates of mosquitoes exposed to untreated papers served as the control for the experiment. Morphological identification of adult mosquitoes was done for *Anopheles* and *Aedes* mosquitoes using the identification keys by Rueda [31] and Coetzee [32] respectively. Further confirmation of species was done by adopting the processes outlined in previous studies [34, 35].

### Piperonyl butoxide (PBO)-Insecticide bioassay

The PBO assay adopted same mosquito populations that were exposed to the various insecticides with confirmed resistance. The experiment was done by pre-exposing the mosquitoes to PBO (4%) for one hour and to the insecticide for which resistance has been reported for another one hour [21]. The exposure was done in four replicates comprised of twenty-five mosquitoes each. Similar steps taken in IRA were done in PBO-insecticide assay [26].

### Statistical analysis

Mortality data were assessed for resistance/susceptibility. Mortality below 90% was considered confirmed resistance, Mortality between 90% and 97% was considered possible resistance and mortality between 98% and 100% was considered susceptibility [21]. Knockdown time was modeled using Probit analysis for 50% and 95% of mosquito population [36]. One-way Analysis of Variance (ANOVA) was done to check for significant difference using XL Stat version 23 [37].

## Results

### Knockdown of male mosquitoes

**Fig 1:**
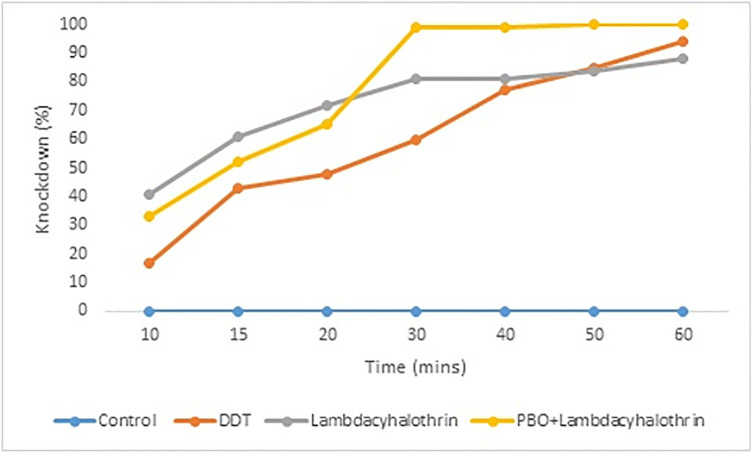
Knockdown record of *Ae*. *aegypti* to DDT and Lambda-cyhalothrin in Aniocha South, Delta State (F_ANOVA_ = 6.605, p = 0.00081).

The knockdown of *Ae*. *aegypti* mosquitoes exposed to DDT was the lowest from 10 to 40 minutes. This was closely followed by knockdown in male mosquitoes exposed to PBO-lambda-cyhalothrin in Aniocha South, Delta State (Fig 1). The knockdown of mosquitoes exposed to lambda-cyhalothrin and PBO-lambda-cyhalothirin met at 30 minutes while that of DDT and lambda-cyhalothrin met at 50 minutes. Complete knockdown was recorded in male mosquitoes exposed to PBO+lambda-cyhalothrin. The differences between the knockdown of mosquitoes were significant (F_ANOVA_ = 6.605, p = 0.00081).

**Fig 2:**
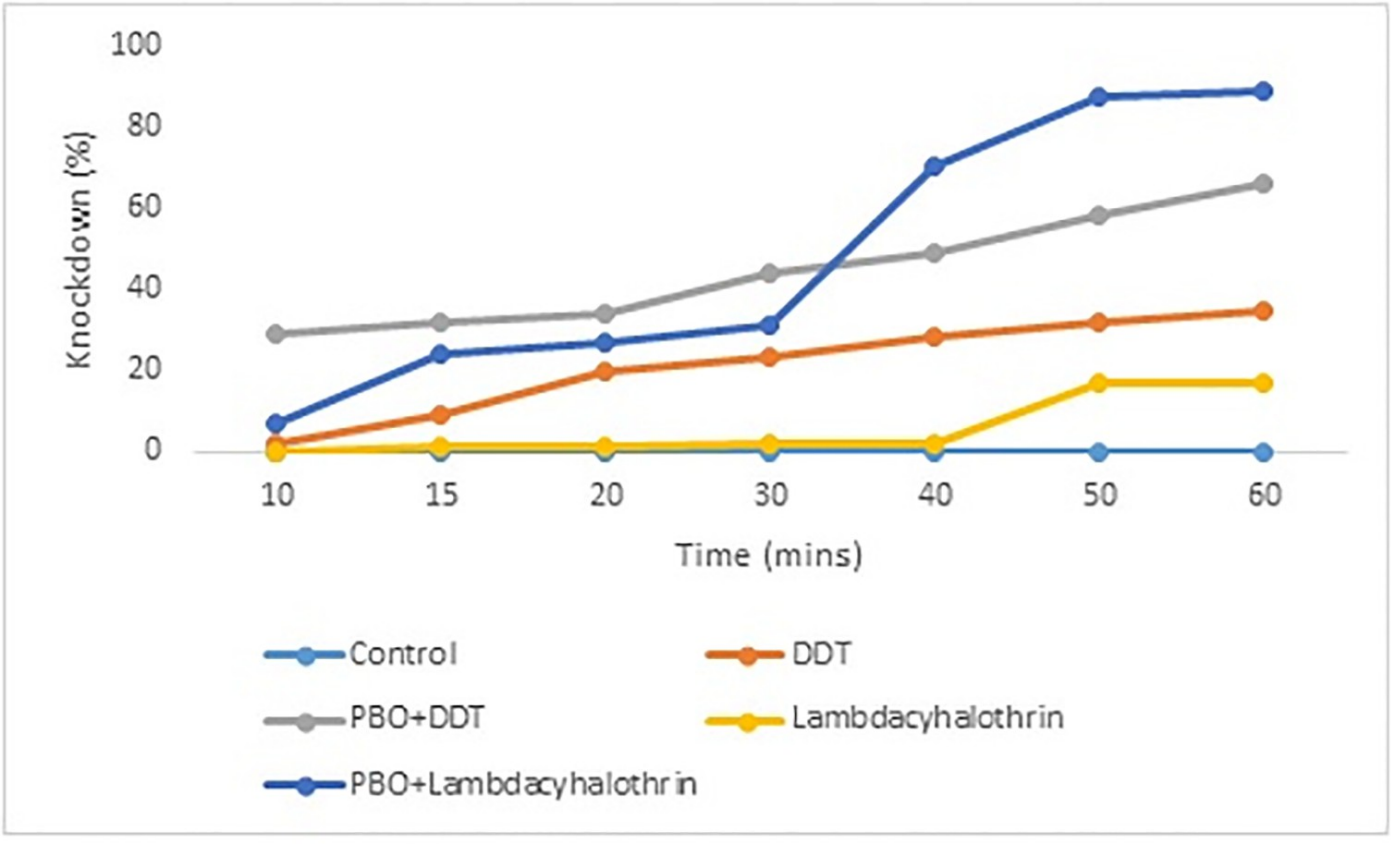
Knockdown record of *An*. *gambiae* to DDT and lambda-cyhalothrin in Aniocha South, Delta State (F_ANOVA_ = 5.066, p = 0.0017).

The knockdown of *An*. *gambiae* mosquitoes to lambda-cyhalothrin was the lowest and was closely followed by DDT exposure (Fig 2). The knockdown was highest in PBO+DDT exposure from 10–30 minutes. The knockdown of male mosquitoes met PBO+DDT exposure at 30 minutes and increased to 89% after 60 minutes compared to PBO+DDT (66%). The differences between the knockdown of mosquitoes were significant (F_ANOVA_ = 5.066, p = 0.0017).

### Knockdown resistance (Kdr) time model

The knockdown resistance time model of mosquitoes exposed to DDT and lambda-cyhalothrin in Aniocha South, Delta State is shown in Table 1. The knockdown time for 50% and 95% in male *An*. *gambiae* mosquitoes exposed to DDT and synergist ranged from 39.5 to 71.2 minutes, and 124.5.71 to 146.4 minutes respectively. The knock down time in same mosquito population exposed to lambda-cyhalothrin and synergist ranged from 33.0 to 81.8 minutes and 64.0 to 124.4 minutes respectively. More so, in *Ae*. *aegypti* male mosquitoes exposed to DDT, KDT 50% and 95% was 23.9 and 61.7 minutes while that for lambda-cyhalothrin and synergist ranged from 5.6 to 15.3 minutes, and 36.1 and 72.3 minutes respectively. The lowest knockdown time was observed in *Ae*. *aegypti* mosquitoes exposed to lambda-cyhalothrin (5.59 minutes). This was closely followed by same mosquitoes exposed to PBO+ lambda-cyhalothrin (15.31 minutes). The highest knockdown time for 50% was recorded in *An*. *gambiae* mosquitoes exposed to lambda-cyhalothrin. Similarly, the highest knockdown time for 95% mortality was recorded in *An*. *gambiae* mosquitoes exposed to DDT. There were significant differences in the knockdown models (p<0.0001).

**Table 1:** Knockdown resistance time model of male mosquitoes exposed to DDT and lambda-cyhalothrin in Aniocha South, Delta State, Nigeria.

Mosquitoes	Insecticide	Regression equation	R^2^ (P-value)	KDT_50_ (95% CI)	KDT_95_ (95% CI)
*An*. *gambiae*	DDT	y = 0.02x-1.56	0.07 (<0.0001)	71.19 (62.19–86.64)	146.39 (121.23–191.82)
	PBO+ DDT	y = 0.02x-0.77	0.05 (<0.0001)	39.50 (34.55–45.70)	124.49 (103.54–161.94)
	Lambda-cyhalothrin	y = 0.04x-3.17	0.18 (<0.0001)	81.84 (72.58–98.87)	124.37 (105.35–160.60)
	PBO+ Lambda-cyhalothrin	y = 0.05x-1.75	0.29 (<0.0001)	32.99 (30.97–35.07)	64.01 (59.81–69.32)
*Ae*. *aegypti*	DDT	y = 0.04x-1.04	0.20 (<0.0001)	23.88 (21.24–26.29)	61.71 (56.72–68.34)
	Lambda-cyhalothrin	y = 0.03x-0.14	0.08 (<0.0001)	5.59 (-3.67–11.62)	72.33 (62.70–88.05)
	PBO+ Lambda-cyhalothrin	y = 0.03x-0.14	0.35 (<0.0001)	15.31 (13.40–16.96)	36.06 (33.13–40.08)

**Note:** N = 100; 50% and 95% Knockdown time, KDT_50_ and KDT_95_, are in minutes; Adjusted R: R^2^ * shows significance at p< 0.05 and ** highly significant at p< 0.0001.

### Resistance assay of male mosquitoes

The resistance bioassay of male mosquitoes to DDT and lambda-cyhalothrin in Aniocha South, Delta State is shown in Table 2. *An*. *gambiae* mosquitoes exposed to DDT and lambda-cyhalothrin were confirmed resistant (61% and 53% respectively). Exposure of same male population to PBO and insecticides showed an increased mortality to 90% in DDT and 98% in lambda-cyhalothrin. Males of *Ae*. *aegypti* mosquitoes were susceptible to DDT but resistant to lambda-cyhalothrin (87%) *Ae*. *aegypti* male mosquitoes exposed to PBO-lambda-cyhalothrin showed complete mortality (100%).

**Table 2:** Resistance bioassay of male mosquitoes to DDT and lambda-cyhalothrin in Aniocha South, Delta State.

Mosquitoes	Insecticide	Mortality after 24 hours (%)	Status
*An*. *gambiae*	DDT	61	Confirmed resistance
	PBO+ DDT	90	Possible resistance
	Lambda-cyhalothrin	53	Confirmed resistance
	PBO+ Lambda-cyhalothrin	98	Susceptible
*Ae*. *aegypti*	DDT	98	Susceptible
	Lambda-cyhalothrin	87	Confirmed resistance
	PBO+ Lambda-cyhalothrin	100	Susceptible

## Discussion

Insecticide resistance, especially to recommended strategies is the major setback to effective vector control intervention. It has remained the significant obstacle for the past decades. A prior understanding on the resistance status of mosquito populations to recommended insecticides adopted for vector control is crucial in informing vector strategies and directing policies for sustainability. Insecticide resistance can be curbed by investigating the status of the vector in lieu of the need to determine the resistance status of mosquitoes causing disease in the various locations. There are no studies reporting the susceptibility status of male mosquitoes to insecticides. Therefore, this study compared the knockdown rate and mortality of mosquitoes to exposed insecticides adopting previous studies on female mosquitoes. This is simply to ascertain the variation in mortality and knockdown rate due to the sex of the species. Temporal and spatial variation in mosquito resistance to recommended insecticides have been reported [38]. The result of the study has revealed that *An*. *gambiae* mosquitoes exposed to DDT and lambda-cyhalothrin were resistant. This finding corroborates several studies; Boussougou-Sambe *et al*. [39] in a province in Gabon whereby resistance to DDT and a pyrethroid was reportedly lower than 10%, Santacoloma *et al*. [40] high resistance of *Anopheles* mosquitoes to DDT and lambda-cyhalothrin in Colombia and high resistance of *Anopheles* to pyrethroids and DDT in the Niger-Delta regions [41]. Likewise, Fagbohun *et al*. [42] reported below 25% mortality in *Anopheles* exposed to DDT in Lagos State. Interestingly, the mortality rates observed in this present study were notably higher than those reported in previous studies, suggesting potentially low insecticide usage in our study area. More so, the high mortality rate in male mosquitoes may be linked to the fact that they do not seek blood meal indoors like the female species and they probably display less contact to insecticide sprayed indoors.

Contrary to the findings with *An*. *gambiae* mosquito exposures, *Ae*. *aegypti* mosquitoes exhibited resistance to lambda-cyhalothrin while remaining susceptible to DDT. This finding aligns with previous studies in Nigeria and abroad, indicating higher resistance of *Ae*. *aegypti* mosquitoes to organochlorine and pyrethroids [43–48]. Mortality as low as 3% in *Aedes* mosquitoes exposed to DDT has been reported by Mukhtar and Ibrahim, [49]. The mosquito resistance reported in these studies portends the likelihood of pyrethroid and DDT usage in the environment. Differences between resistance patterns within the *Aedes* complex is common and this highlights the need for tailored molecular investigation to unravel the specific genes responsible. *Ae*. *aegypti* mosquitoes in this study was reportedly resistant to lambda-cyhalothrin and not to DDT. This finding is not in line with the study of Demok *et al*. [46], whereby *Aedes albopictus* mosquitoes were susceptible to pyrethroids. The subspecies differences in resistance to pyrethroids between *Anopheles* mosquitoes were equally observed by Mawejje et al. [50]. The complex nature of resistance in mosquitoes due to locational differences can be best explained from anthropogenic activities and environment. Households in Nigeria use insecticide treated nets, apply insecticides for various activities and equally adopt insecticides as last resort for vector control which probably could have bio-accumulated, biomagnified and passed to the mosquitoes from the environment. A study by Wilson *et al*. [1] has acknowledged the use of insecticides in the efficient control of mosquitoes determined through the knock down rate and eradication of mosquito-borne diseases. The mortality recorded in this study could be best explained following the molecular mechanisms described by Bharati and Saha, [51], Tchigossou et al. [52], Djiappi-Tchamen et al. [53], and Muhammad et al. [41]. The explanation made in previously published findings is probably linked to the observations made in this present study during pyrethroid exposures which pointed that kdr mutation and environmental factor could be responsible to the low mortality rate in *An*. *gambiae* mosquitoes exposed to the various insecticides.

This study equally evaluated the effectiveness of synergists in breaking the barrier to mosquito susceptibility. Pre-exposure to synergists partially or completely restored susceptibility in both *An*. *gambiae* and *Ae*. *aegypti* mosquitoes. In this present study, possible resistance was observed in *An*. *gambiae* exposed to DDT+PBO. However, lambda-cyhalothrin synergized PBO of same *Anopheles* species was susceptible. Lambda-cyhalothrin+PBO in *Aedes* mosquitoes recorded susceptibility. These findings align with previous studies indicating the potentiality of PBO synergized-insecticide in enhancing insecticide efficacy. The increase in mortality due to pre-exposure to PBO has been reported and corroborates this present study [41]. Increased knockdown time rate in PBO-synergized assays substantiate the activity of P450s and the basic need for actions. Combination of PBO and pyrethroids in several trials have proven to provide valuable improvements in the reduction of vector density and diseases transmission [22, 25]. Further explanation for the possible reasons for no high resistance in mosquitoes exposed to the pyrethroid and DDT in this location may be due to that the trace of DDT in the environment is limited and mosquitoes are probably not pre-exposed to the insecticide. It is probable that farmers do not use this insecticide for farming, absence of river for fishing and that these mosquitoes even though day biting are more common around the farm than in the house.

Analysis of knockdown dynamics revealed interesting patterns in resistance mechanisms. Anopheles mosquitoes exposed to synergized DDT exhibited increased knockdown rates, suggesting metabolic resistance mechanisms. High significant knockdown of *An*. *gambiae* mosquitoes exposed to DDT and synergist was equally observed. The knockdown trend observed in this study is not in accordance with the study of Kampango *et al*. [48] where no increase was observed in synergized insecticides. This simply implicate the involvement of various enzymes for resistance as earlier reported [21, 50, 51]. It can therefore be inferred that the absence of significant resistance in mosquitoes exposed to pyrethroids and DDT may be attributed to limited environmental exposure to these insecticides. Factors such as agricultural practices and mosquito habitat preferences may influence insecticide exposure levels and resistance development.

## Conclusion

This study shows that *An*. *gambiae* mosquitoes were highly resistant to DDT and mortality increasing to 90% when combined with synergist. Complete mortality was observed for *Ae*. *aegypti* mosquitoes exposure to DDT. This implies that these insecticides are suitable and effective in the control of male mosquitoes in the location. It equally underscores the importance of monitoring insecticide resistance patterns in mosquito populations and the potential of PBO synergized insecticides in breaking the barrier created by resistance. Strategies targeted towards the effective control of male mosquitoes can still utilize these insecticides. However special care is required to understudy the potential role of male in transferring resistant genes to offspring.
