# Correction: Susceptibility status and synergistic activity of DDT and Lambda-cyhalothrin on *Anopheles gambiae* and *Aedes aegypti* in Delta State, Nigeria

**DOI:** 10.1371/journal.pone.0328385

**Published:** 2025-07-15

**Authors:** Chioma C. Ojianwuna, Victor N. Enwemiwe, Eric Esiwo, Favour Mekunye, Ann Anigbobi, Treasure E. Oborayiruvbe

The fifth author’s name is spelled incorrectly. The correct name is: Ann Anigbobi. The correct citation is: Ojianwuna CC, Enwemiwe VN, Esiwo E, Mekunye F, Anigbobi A, Oborayiruvbe TE (2024) Susceptibility status and synergistic activity of DDT and Lambda-cyhalothrin on *Anopheles gambiae* and *Aedes aegypti* in Delta State, Nigeria. PLoS ONE 19(8): e0309199. https://doi.org/10.1371/journal.pone.0309199.
